# Mechanisms of Hepatocellular Injury in Hepatitis A

**DOI:** 10.3390/v13050861

**Published:** 2021-05-08

**Authors:** Minghang Wang, Zongdi Feng

**Affiliations:** 1 Center for Vaccines and Immunity, The Abigail Wexner Research Institute at Nationwide Children’s Hospital, Columbus, OH 43205, USA; minghang.wang@nationwidechildrens.org; 2 Department of Pediatrics, The Ohio State University College of Medicine, Columbus, OH 43210, USA

**Keywords:** hepatitis A virus, liver injury, bystander T cell activation, MAVS signaling, TIM-1 polymorphism, IL-18BP deficiency

## Abstract

Hepatitis A virus (HAV) infection is a common cause of acute viral hepatitis worldwide. Despite decades of research, the pathogenic mechanisms of hepatitis A remain incompletely understood. As the replication of HAV is noncytopathic in vitro, a widely accepted concept has been that virus-specific cytotoxic T cells are responsible for liver injury. However, accumulating evidence suggests that natural killer (NK) cells, NKT cells, and even non-HAV-specific CD8^+^ T cells contribute to liver damage during HAV infection. In addition, intrinsic death of virus-infected hepatocytes has been implicated as a cause of liver injury in a murine model of hepatitis A. Furthermore, genetic variations in host factors such as T cell immunoglobulin-1 (TIM1) and IL-18 binding protein (IL-18BP) have been linked to hepatitis A severity. This review summarizes the current knowledge of the mechanisms of hepatocellular injury in hepatitis A. Different mechanisms may be involved under different conditions and they are not necessarily mutually exclusive. A better understanding of these mechanisms would aid in diagnosis and treatment of diseases associated with HAV infection.

## 1. Introduction

Hepatitis A virus (HAV) infection is a common cause of acute viral hepatitis worldwide. HAV is transmitted via a fecal–oral route and is most prevalent in resource-poor countries where transmission via contaminated food or water often causes point source outbreaks [1]. HAV infection is usually self-limited, but can cause debilitating disease and even death [2]. Despite the existence of highly effective vaccines, an estimated 47 million infections occurred in 2010, resulting in 94,000 deaths [3]. Although outbreaks in industrialized countries are relatively uncommon, the most recent outbreak of hepatitis A in the United States started in 2016, involved 35 states, and signaled the re-emergence of hepatitis A. As of April 16, 2021, there are 38,795 publicly reported cases, resulting in 23,585 hospitalizations (61%) and 372 deaths (https://www.cdc.gov/hepatitis/outbreaks/2017March-HepatitisA.htm, accessed on 1 April 2021).

After fecal–oral transmission of HAV, the virus enters the blood stream via a poorly understood process to gain access to the liver, the target organ, for its propagation. Acute HAV infection is associated with a lengthy incubation period (4–6 weeks), during which large amounts of virus are shed into the feces [1]. The virus is detected in the blood (viremia) for several weeks before and after the onset of clinical symptoms. During the peak of virus shedding, most patients produce HAV-specific IgM, followed by IgG. The appearance of anti-HAV IgM coincides with elevated liver enzymes [Alanine aminotransferase (ALT) and aspartate aminotransferase (AST)] in the serum, a hallmark of liver damage. Cytotoxic T cells are detected in the acute phase and early convalescent phase and play a pivotal role in viral clearance [4].

HAV is a small RNA virus belonging to the *Picornaviridae* family. Unlike many other picornaviruses, HAV replicates very slowly and does not cause apparent cytopathic effects in cell culture [5]. This is in line with a relatively long incubation period of acute HAV infection in humans [1,4]. Our recent work has shown that HAV usurps the cellular endosomal sorting complex to exit as membrane-cloaked, quasi-enveloped particles (eHAV), providing a long-sought explanation for noncytolytic release and spread of HAV [6]. The eHAV particles are the only virion form detected in patient serum, thus they most likely mediate the virus spread to the liver [7].

Studying HAV pathogensis in patients has been difficult due to limited access to patient samples. Several species of non-human primates (NHPs), including mamosets, owl monkeys, and chimpanzees, are susceptible to HAV and produce diseases similar to those in humans, therefore have been used for studying the pathogenesis associated with HAV infection [8,9]. More recently, a murine model was established by Lemon and colleagues [10]. Studies in these animal models have provided insights into the pathogensis of HAV infection in humans.

## 2. Clinical Features of Hepatitis A

Symptoms associated with hepatitis A are clinically indistinguishable from those of other types of viral hepatitis. Typically, HAV infection results in acute viral hepatitis characterized by fatigue, fever, nausea, vomiting, anorexia, abdominal pain, and jaundice [11,12]. ALT and AST concentrations in patient serum are considered sensitive indicators of hepatocellular injury [13], providing reliable quantitative measures of liver damage. Unlike other forms of viral hepatitis, hepatitis A has no documented chronic cases. The clinical outcomes of hepatitis A are generally variable, ranging from asymptomatic to fulminant hepatitis [11,14]. In general, children with hepatitis A experience a much milder clinical course than adults; about 70% of HAV infection in children are asymptomatic, while symptomatic infection rates in adults are estimated to be higher than 70% [15,16]. Fulminant hepatitis, an unusual complication of hepatitis A, is considered a significant contributor to death in patients [17]. 

Hepatitis A has multiple atypical manifestations, including relapsing hepatitis, cholestatic hepatitis, and extrahepatic manifestations [18,19,20]. Relapsing hepatitis usually occurs 4 to 15 weeks following acute viral hepatitis. A much milder clinical development in the relapsing phase of hepatitis A is generally observed than in the initial infection [21,22]. However, patients may experience more than one relapsing course of hepatitis A [22]. The mechanism underlying the recurrence of hepatitis A remains poorly understood. HAV-specific IgA has been proposed to serve as a carrier to facilitate HAV transport to the liver, thereby contributing to relapsing hepatitis A [23]. However, the significance of this finding is questionable because circulating eHAV particles unlikely bind to IgA. As for cholestatic hepatitis, it is usually marked by pruritus, fever, diarrhea, and weight loss, along with high levels of bilirubin in patient serum [19]. Furthermore, HAV infection is associated with multiple extrahepatic manifestations, including acute kidney injury, autoimmune hemolytic anemia, aplastic anemia, and acute pancreatitis [11,20]. However, these manifestations are rare.

## 3. Histologic Features of Hepatitis A in Humans

Microscopic changes in HAV infection have been extensively investigated by analyzing liver biopsy specimens collected from patients with hepatitis A. Acute hepatitis A displays both degenerative and regenerative parenchymal changes, along with infiltrating inflammatory cells [22]. However, the composition and proportion of inflammatory cells in acute hepatitis A have not been well established. There is significant lobular disarray characterized by swollen hepatocytes and hepatocyte necrosis, especially in the centrilobular and midzonal areas. Kupffer cells in the sinusoids are activated and marked by hypertrophy and hyperplasia. The portal tracts are enlarged and infiltrated by lymphocytes, neutrophils, eosinophils, and plasma cells [22,24]. Of note, varying degrees of portal fibrosis also occur in some cases [24]. The limiting plate of hepatocytes is disrupted due to periportal changes. Notably, piecemeal necrosis marked by bridging hepatic necrosis, which is considered more common in chronic hepatitis, may also befall some patients with acute hepatitis A [24,25]. 

The morphological features in patients with acute hepatitis A may vary depending on age, gender, disease severity, and the time of liver tissue harvesting [25,26,27,28]. In patients with fulminant hepatitis, massive hepatic necrosis takes over the liver [29]. The massive loss of hepatocytes is usually accompanied by accumulation of inflammatory cells, including T cells, NK cells, B cells, and both M1 and M2 macrophages [30]. In cholestatic hepatitis, there is a varying degree of cholestasis characterized by bile thrombi, cholestatic rosettes, and ductular transformation of hepatocytes [31]. Serology tests are usually employed to determine the causes of acute viral hepatitis because patients with acute hepatitis A, B, C, or E infection exhibit similar histologic features in liver tissues. Nevertheless, liver biopsy remains the gold standard for assessing the severity of liver injury.

## 4. Mechanisms of Liver Injury during HAV Infection

Acute HAV infection has a stealthy nature. The virus replicates logarithmically in the liver without any symptoms. Studies with experimentally infected chimpanzees revealed a very limited induction of type I interferon-stimulated genes in the liver during the acute phase [9]. This blunted type I IFN response is attributable to HAV protease-mediated cleavage of the mitochondrial antiviral signaling protein (MAVS) and TIR-domain-containing adaptor-inducing interferon-β (TRIF) [32,33]. These two proteins are adaptors in the cytoplasmic viral RNA sensing pathways. HAV is sensitive to type I IFN in vitro, thus its ability to limit IFN production in infected cells is likely critical for its survival. The long-term persistence of intrahepatic viral RNA in HAV-infected chimpanzees may also be relevant to relapsing hepatitis seen in up to 20% of patients following apparent resolution of acute hepatitis [9].

The pathogenesis of hepatitis A and the mechanisms underlying hepatocellular injury are incompletely understood. Since HAV does not directly cause cytopathic effects, it is unlikely that viral cytolysis is responsible for liver injury during acute HAV infection. Histological findings in liver biopsies obtained from patients and experimentally infected animals demonstrate a strong temporal correlation between immune cell infiltration and the disease, suggesting that cell-mediated mechanisms are critical for liver damage. While virus-specific cytotoxic T lymphocytes (CTLs) have been generally thought to be a major driver for HAV-associated immunopathology, emerging evidence suggests additional mechanisms are involved. Below we summarize the current knowledge of the mechanisms involved in liver injury caused by HAV infection. 

### 4.1. Virus-Specific Cytotoxic T Lymphocytes (CTLs) 

**Figure 1:**
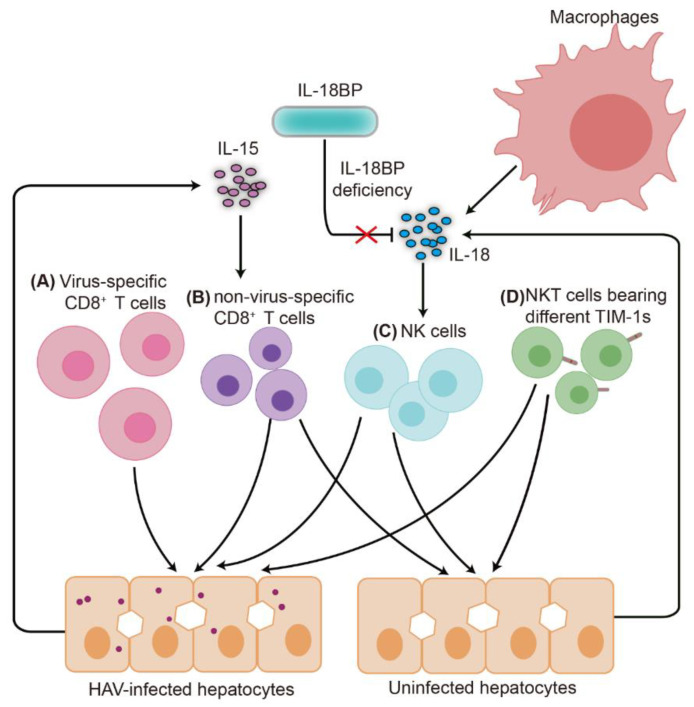
Proposed mechanisms of liver injury mediated by HAV. (**A**) During HAV infection, the virus activates CD8^+^ T cells, generating virus-specific CD8^+^ T cells. Activated virus-specific CD8^+^ T cells are differentiated into effector cytotoxic T lymphocytes that specifically kill virus-infected cells, thus contributing to liver injury. (**B**) In patients with hepatitis A, high levels of IL-15 in the serum activate non-virus-specific CD8^+^ T cells, which are capable of lysing both infected and uninfected hepatocytes. (**C**) High levels of IL-18 have been detected in both macrophages and hepatocytes in IL-18BP-deficient-patients with fulminant hepatitis A. Due to lack of neutralizing activity against IL-18, excessive and uncontrolled IL-18 activates NK cells, which subsequently mediate the lysis of both infected and uninfected hepatocytes. (**D**) In patients with severe hepatitis A, HAV seems to activate NKT cells in a TIM-1 dependent manner. HAV-infected cells had higher cytotoxic activity in NKT cells carrying the longer form of TIM-1 than in NKT cells harboring the wild type TIM-1, thereby contributing to liver injury. Apoptosis of HAV-infected hepatocytes mediated by MAVS-IRF3/IRF7-dependent signaling has also been implicated in liver injury in a murine model of HAV [10].

Virus-specific CTLs play a pivotal role in clearing virus-infected cells. However, they may also cause tissue damage, thereby contributing to pathogenesis. Using peripheral blood lymphocytes (PBLs) and autologous skin fibroblasts from patients with hepatitis A, Vallbracht and colleagues demonstrated that PBLs could lyse HAV infected skin fibroblasts in a human leukocyte antigen (HLA)-dependent manner [34,35]. The highest cytolytic activity was found with PBLs collected during the early convalescent phase. However, it is possible that a significant portion of the cytolytic cells accumulates in the liver at the climax of the disease [34]. A follow-up study was conducted by the same group to confirm that liver-derived CD8^+^ T cells were able to lyse HAV-infected skin fibroblasts [36]. These initial studies provided the first evidence that virus-specific CTLs are involved in mediating liver injury in acute hepatitis A (Figure 1A). 

In 2011, Schulte et al. analyzed CD8^+^ T cells in patients with acute, post-acute, and resolved HAV infection using class I multimers [37]. A broad CD8^+^ T cell response was found during the acute phase of HAV infection. In the same year, Yan et al. performed a detailed analysis of T cell responses in chimpanzees experimentally infected with HAV [38]. In contrast to the Schutle study, the CD8^+^ T cell response was not found to be temporally associated with control of infection or liver damage. Instead, a dominant CD4^+^ T cell response was associated with virus clearance. This pattern was observed for T cells both in blood and in liver [38]. Of note, transaminase elevations were milder in the two HAV-infected chimpanzees than in symptomatic human infections. Thus, it remains to be determined if CD8^+^ T cells exert a species-specific role or play a more important role in severe liver disease. 

### 4.2. Liver Damage Mediated by Non-HAV-Specific Lymphocyte Activation

Accumulating evidence suggests that non-virus-specific lymphocytes also contribute to liver injury in hepatitis A. As mentioned above, NK cells can lyse K562 cells and HAV-infected cells in a non-specific manner in vitro [34]. Interestingly, PBLs from healthy donors never exposed to HAV exhibited greater lytic capability for HAV-infected BS-C-1 cells than for uninfected cells [39]. The cellular composition involved in the lysis of HAV-infected cells was determined to be positive for CD16^+^ and CD11b^+^ markers similar to those in NK cells. These data indicated that cytotoxicity mediated by non-HAV-specific lymphocytes such as NK cells likely contributed to the lysis of HAV-infected cells. It should be noted that the effector cells in this study came from humans, while the target cells were derived from the African Green Monkey kidney. Whether the results could reflect the mode of action of PBLs against HAV-infected cells remains uncertain. 

In addition to NK cells, non-HAV-specific CD8^+^ T cells have been shown to contribute to liver injury [40]. In patients with hepatitis A, non-HAV-specific CD8^+^ T cells were activated by high levels of IL-15, which was referred to as “bystander activation” [40]. These bystander-activated CD8^+^ T cells were capable of lysing Huh-7 cells. The cytolytic activity of these CD8^+^ T cells could be blocked by antibodies against natural killer cell activating receptors, such as NKG2D and NKp30. Of note, NKG2D ligands, including MIC-A and MIC-B, were readily detected in HAV-infected and uninfected hepatocytes, leading to the conclusion that both HAV-infected and uninfected hepatocytes may serve as targets for non-HAV-specific CD8^+^ T cells [40] (Figure 1B). A more recent study by El Costa et al. described similar results in hepatitis E virus (HEV)-infected-patients [41], suggesting that the involvement of non-virus-specific CD8^+^ T cells in liver damage is probably more common than previously appreciated.

### 4.3. Emerging Role of Intrinsic Apoptosis in HAV-Induced Liver Damage

In 2016, Hirai-Yuki et al. described the development of a murine model for HAV infection [10]. HAV does not infect mice but replicates efficiently in mice deficient in the type I IFN receptor (IFNAR1) or MAVS. Interestingly, IFNAR1-deficient mice developed hepatitis after HAV infection, but MAVS-deficient mice did not. Typical histopathologic lesions, including necrotic or apoptotic hepatocytes and inflammatory cell infiltration, were observed in their liver tissue. However, these typical histopathologic lesions were not observed in MAVS-deficient mice, although HAV replicated 10 times more efficiently in the MAVS-deficient mice than in the IFNAR1-deficient mice. The results in IRF3- or IRF7-deficient mice were similar. Notably, IFNAR1-deficient mice had low-level virus-specific T cell responses, suggesting that the HAV-mediated liver injury in mice was related to the MAVS-IRF3/IRF7 signaling pathway but not CTLs. Further supporting this notion, depletion of T cells had little effect on HAV-induced ALT elevation in IFNAR-deficient mice. However, the precise mechanism involving MAVS-mediated apoptosis in HAV-induced liver injury remains unknown.

A separate study has shown that HAV can robustly infect Alb-uPA/SCID mice engrafted with human hepatocytes [42]. In this model, mice with severe combined immunodeficiency were engineered to express a urokinase-type plasminogen activator (uPA) under the albumin promoter. The high-level expression of uPA resulted in progressive death of murine hepatocytes, allowing their replacement with transplanted human hepatocytes. The lack of adaptive immunity in these mice offered a unique advantage to assess HAV-induced liver damage in the absence of adaptive immune responses, although ongoing hepatotoxicity due to transgene expression might have been a confounding factor. Similar to HAV-infected chimpanzees, a minimal type I and type II IFN response was detected in the HAV-infected humanized mice. No definite evidence for hepatocyte damage related to HAV infection was found in that study [42].

These studies with murine models have provided fresh insights into HAV infection and pathogenesis. A surprising finding is that MAVS-mediated signaling not only determines the host species range of HAV but also results in intrinsic apoptosis of HAV-infected hepatocytes. These data imply that cleavage of MAVS by HAV protease not only enhances virus replication but may also help prevent/reduce liver damage. Whether these observations recapitulate the actual events of HAV infection in humans merits further study.

### 4.4. Host Genetic Factors Involved in the Severity of Hepatitis A

Several host genetic factors have been shown to be linked to the severity of hepatitis A. A patient with fulminant hepatitis presented with a 40 nt homozygous deletion in the IL-18 binding protein (IL-18BP) gene generating three novel splice variants of IL-18BP [30]. Intriguingly, all three isoforms of IL-18BP were prone to be degraded and lacked neutralizing activity against IL-18, thus resulting in complete IL-18BP deficiency. Patients with fulminant hepatitis A also had a high level of IL-18 in both macrophages and hepatocytes. Consequently, both HAV-infected and uninfected hepatocytes were killed by NK cells due to their activation by excessive and uncontrolled IL-18 production [30]. Therefore, uncontrolled IL-18–mediated cytotoxic activity against hepatocytes was likely responsible for liver destruction in this particular patient [30] (Figure 1C). 

Of note, IL-18 has been associated with susceptibility and disease progression of other forms of viral hepatitis, including hepatitis B [43,44,45], hepatitis C [46,47,48], and hepatitis E [41,49]. For example, the HBV X protein is able to upregulate IL-18 expression, which in turn upregulates FasL expression in hepatoma cells [43]. Recombinant IL-18 inhibits HBV replication in HBV transgenic mice by activating intrahepatic NK cells and NKT cells and by inducing type I IFN production in infected liver [44]. IL-18, TNF, and IFN-γ alleles and genotypes are also associated with susceptibility to chronic hepatitis B infection and severity of liver injury [45]. In patients with hepatitis E, IL-18 activates non-HEV-specific effector memory CD8^+^ T cells, thereby contributing to HEV pathogenesis [41]. IL-18 has also been associated with HEV-induced renal disorder [49]. Thus, IL-18–mediated cytotoxic activity appears to be a common contributing factor to liver injury in viral hepatitis, and the genetic variations in IL-18/IL-18BP likely may have a significant impact on host susceptibility and/or the severity of the disease.

An epidemiological study of children with severe hepatitis A identified a 6-amino-acid insertion in T-cell immunoglobulin and mucin domain 1 (TIM-1) as a genetic factor associated with the severity of hepatitis A [50]. TIM-1 was initially discovered as a cellular receptor for HAV [51,52]. In patients with severe hepatitis A, NKT cells seem to be activated by HAV in a TIM-1 dependent manner. A longer TIM-1 form displayed higher binding affinities to HAV particles than the wild-type TIM-1. Correspondingly, NKT cells carrying a longer TIM-1 form had a higher cytotoxic activity for HAV-infected cells [50]. Thus, the authors proposed genetic variations in TIM-1 as a mechanism responsible for the severity of HAV-induced hepatitis (Figure 1D). However, this model was questioned by Das and colleagues, who demonstrated that TIM-1 is not essential for cellular entry of HAV [53]. Overall, both IL-18BP and TIM-1 genes are biologically plausible susceptibility genes for hepatitis A, facilitating a risk evaluation for developing severe viral hepatitis in patients.

Although not discussed in detail, viral factors likely also play a role in modulating the disease process. For example, the HAV protease processing intermediates 3ABC and 3CD cleave the multiple cellular factors MAVS and TRIF, respectively [32,33], thereby dampening the innate response to infection. HAV also circulates in the bloodstream as quasi-enveloped virions which are not recognized by circulating anti-HAV antibodies but can activate plasmacytoid dendritic cells to produce type I interferons [54]. Finally, genetic variations among HAV genotypes and strains could affect virus-host interactions and disease pathogenesis, an area that so far has not been carefully studied.

Collectively, these new studies suggest that multiple components of the immune system as well as genetic variations in certain host factors play roles in hepatocellular damage during HAV infection. These mechanisms are not necessarily mutually exclusive, and it is likely that either one or multiple mechanisms are involved in different disease conditions. While many of these observations described above await validations by independent studies, they nonetheless provide a framework for further investigation of the mechanisms involved in HAV pathogenesis. More integrated approaches may be needed for better understanding the mechanisms that drive liver damage in patients. 

## 5. Conclusions

Despite decades of research, the mechanisms of liver injury in hepatitis A remain incompletely understood. While virus-specific CD8^+^ T cells have long been considered a major cause of HAV-induced liver damage, new evidence suggests that additional mechanisms are involved. Other immune cells, including non-HAV-specific CD8^+^ T cells, NK cells, and NKT cells, have been shown to contribute to liver damage. Additionally, intrinsic apoptosis mediated by MAVS signaling appears to be responsible for hepatitis in a murine model of HAV infection. Furthermore, genetic variations in both host and viral factors can also influence the severity of hepatitis A. These mechanisms are not mutually exclusive and can act either alone or in combination to drive liver damage in patients. It is almost certain that with the ever-increasing knowledge and advances in technologies, additional mechanisms will be discovered. Further elucidation of these mechanisms of HAV-induced hepatocellular injury will shed new light on the pathogenesis of hepatitis A and facilitate discovering novel therapeutic approaches.
